# Supplementary material for: The Evolution and Characterization of the RNA Interference Pathways in Lophotrochozoa
Source: Genome Biol Evol. 2024 May 7;16(5):evae098. doi: 10.1093/gbe/evae098 (PMC11114477; doi:10.1093/gbe/evae098)
Supplement: evae098_Supplementary_Data [file evae098_supplementary_data.zip › Supplementary_materials.zip!/selected_miRNAs/NoGe_selected_miRNAs/mirdeep_pdf_files/NMRB01000267.1_Notospermus_geniculatus_strain_Ushimado_scaffold267,_whole_genome_shotgun_sequence_8670.pdf]

Provisional ID : NMRB01000267.1_Notospermus_geniculatus_strain_Ushimado_scaffold267,_whole_genome_shotgun_sequence_8670

Score total	:	46
Score for star read(s)	:	3.9
Score for read counts	:	41.4
Score for mfe	:	1.2
Score for randfold	:	0
Score for cons. seed	:	-0.6
Total read count	:	93
Mature read count	:	50
Loop read count	:	0
Star read count	:	43

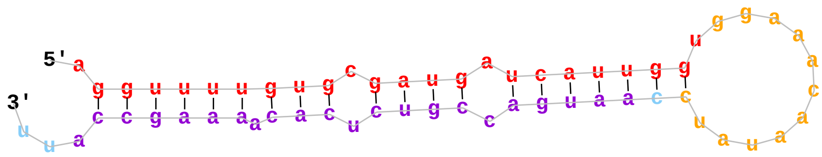

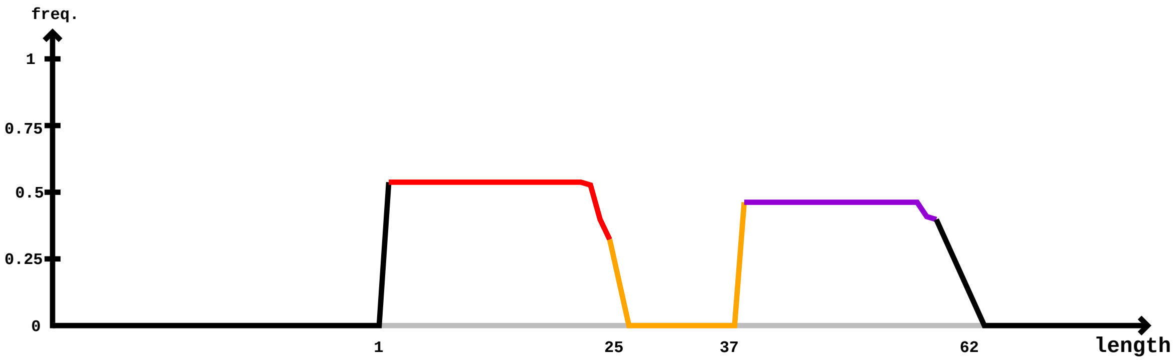

Mature Star

```
5'- ccguguguucaacaaggaaaacguucguagauuagguuuugugcgaugaucauugguggaaacaauauccaaugaccgucucacaaaagccauuucugagcgucaaacaucg  -3'   obs
    ccguguguucaacaaggaaaacguucguagauuagguuuugugcgaugaucauugguggaaacaauauccaaugaccgucucacaaaagccauuucugagcgucaaacaucg         exp
    ((.(((.....))).))...(((((((.((((..(((((((((.((((.(((((((............))))))).)))).))).)))))))))).))))))).........   reads   mm   sample
    .................................agguuuugugcgaugaucauug.........................................................   5       0    Fm3
    .................................agguuuugugcgaugaucauugg........................................................   4       0    Fm3
    .................................agguuuugugcgaugaucauuggA.......................................................   2       1    Fm3
    ......................................................................aaugaccgucucacaaaag.......................   5       0    Fm3
    ......................................................................aaugaccgucucacaaaagc......................   1       0    Fm3
    ......................................................................aaugaccgucucacaaaagcc.....................   1       0    Fm3
    ......................................................................aaugaccgucucacaaaagcca....................   6       0    Fm3
    ......................................................................aaugaccgucucacaaaagccau...................   2       0    Fm3

    .................................agguuuugugcgaugaucauu..........................................................   1       0    Fm1
    .................................agguuuugugcgaugaucauug.........................................................   6       0    Fm1
    .................................agguuuugugcgaugaucauugg........................................................   2       0    Fm1
    ......................................................................aaugaccgucucacaaaagcc.....................   2       0    Fm1
    ......................................................................aaugaccgucucacaaaagcca....................   14      0    Fm1
    ......................................................................aaugaccgucucacaaaagccauu..................   1       0    Fm1

    .................................agguuuugugcgaugaucauuggu.......................................................   1       0    Fm2
    .................................agguuuugugcgaugaucauuggA.......................................................   25      1    Fm2

    .................................agguuuugugcgaugaucauuggu.......................................................   1       0    Mm3
    .................................agguuuugugcgaugaucauugguU......................................................   1       1    Mm3
    ......................................................................aaugaccgucucacaaaagcc.....................   1       0    Mm3
    ......................................................................aaugaccgucucacaaaagcca....................   3       0    Mm3

    .................................agguuuugugcgaugaucauug.........................................................   1       0    Mm2
    .................................agguuuugugcgaugaucauugg........................................................   1       0    Mm2
    ......................................................................aaugaccgucucacaaaagcc.....................   1       0    Mm2
    ......................................................................aaugaccgucucacaaaagcca....................   6       0    Mm2
```
